# Comparative Study of Cerebrospinal Fluid Evaluation in Patients With Acute Leukemia Using the Manual Method and Sysmex XN-1000

**DOI:** 10.7759/cureus.72423

**Published:** 2024-10-26

**Authors:** Mohammed Bensalah, Hasnae Lekfif, Abdelilah Berhili, Assya Khermach, Rachid Seddik

**Affiliations:** 1 Hematology Laboratory, Faculty of Medicine and Pharmacy of Oujda, Mohammed First University, Oujda, MAR; 2 Hematology Laboratory, Mohammed VI University Hospital, Oujda, MAR

**Keywords:** acute leukemia, cerebrospinal fluid, comparative study, cytology, manual method, standardization, sysmex xn-1000®

## Abstract

Background

Many pathological settings can be provided by the cytological analysis of cerebrospinal fluid (CSF). In our work, we aimed to evaluate the concordance between the analysis using the reference manual method and the analysis using Sysmex XN-1000^®^.

Methods

In our study, we examined 121 CSF samples. To count cells in different samples, we used two methods: a manual method using Fast Read 102^®^ counting chambers performed by at least two experienced hematologists and Sysmex XN-1000^®^. Evaluation of the interchangeability and concordance between the two methods was possible thanks to Passing-Bablok regression and Bland-Altman graphs.

Results

Our study showed no evidence of concordance between the manual method and the automated method using Sysmex XN-1000^® ^in cell counting on CSF samples.

Conclusion

Every clinical laboratory aims to use automated and standardized analysis methods in counting and differentiating cells, reducing response time and interlaboratory variability. Our study shows an absence of evidence of concordance between the manual and automated methods using Sysmex XN-1000 in cell counting on CSF samples.

## Introduction

In many cases, information related to the diagnosis and monitoring of many diseases can be provided by the analysis of biological fluids. Among the analysis types of biological fluids, there is the cytological study that determines the number of red blood cells (RBC) and white blood cells (WBC), and there is the differentiation between different WBC types.

Cell count analysis of cerebrospinal fluid (CSF) samples helps in the diagnosis of several inflammatory, infectious, and malignant diseases and allows the classification of patients in different stages of the disease, including RBC counts, which are important for the exclusion of traumatic puncture [1-3].

The traditional method of using a manual hemocytometer (counting chamber) is often used for this purpose. However, in recent years, automated analyzers have become increasingly available for counting cells in body fluids; there are currently many commercialized automated systems enabling cell counting and differentiation in different biological fluids [4,5]. For some types of fluids, automated BF cell-counting analyzers offer accurate results in a short time and reduce inter-observer variability [6]. However, this method has still limited use in cell counting in CSF samples because of their inaccuracy in the low cell concentration ranges [7].

In our laboratory, we performed cell counting by microscopic observation of CSF in Fast Read 102 ® counting chambers. The counting results are expressed in number per µL for both red and white blood cells. The manual method is considered to be a reference method, but it has a number of limitations including the inter-examiner reliability, long delays in getting results, and most importantly, the existence of inter-observer variations [8].

Therefore, the automated method is a solution for these limitations because it provides rapid results (higher throughput), better traceability (connection to the laboratory's computer system), less risk of errors (manual transcription errors), and optimized time for technicians (reduction of repetitive tasks). Another advantage of such systems is the possibility to compare the results of different centers [9].

Before opting for the automated solution as a routine tool, we sought to check the performance of the Sysmex XN-1000® against the microscopic technique.

## Materials and methods

The manual and automated methods were compared by analyzing a total of 121 CSF samples received in the Hematology Unit, Central Laboratory of Mohammed VI University Hospital in Oujda. Our study spanned a period of two years, from December 2019 to December 2021. Most CSF samples were received from the internal medicine and pediatrics departments in order to look for blasts. CSF samples were collected and transported in sterile tubes without anticoagulants. The different samples were assessed within the first hour after receiving the tubes in the laboratory. Samples with insufficient quantity were excluded from the study.

A first cell count (red and white blood cells) was performed by the manual method using Fast Read 102 ® counting chambers (Biosigma S.p.A., Italy), which were analyzed with a light microscope. Cells are counted and reported for a volume of 1 μl. The results of this manual counting were obtained after calculating the mean of the counts from two experienced cytologists. Then a second automatic count using a Sysmex XN-1000® was performed. Sysmex XN-1000® (Sysmex Corporation, Kobe, Japan) is an automated hematological analyzer first released in 2011. For red blood cell counting (as for the platelet counting), it uses the hydrodynamically focused impedance technology, whereas for WBC counting and differentiation, it uses the fluorescence flow cytometry principle via three channels: white cell differential, white cell nucleated, and white cell precursor channels.

To evaluate the concordance between the two methods, we have used Passing-Bablok regression analysis. Statistical significance was based on the 95% confidence intervals (CI): a significant proportional or constant bias was noted when the CI of the slope did not encompass 1 and the CI of the intercept did not encompass 0, respectively. These analyses were completed using the Bland and Altman plot.

The data obtained were unloaded in Excel and analyzed using the statistical software MedCalc Version 15.1.0 (MedCalc Software Ltd., Ostend, Belgium). Then, they were compared using the Passing-Bablok regression model which does not require any particular assumptions concerning the distribution of the samples and measurement errors. The Bland-Altman diagram was also used where the differences between the two techniques were plotted against the means of the two techniques [10,11].

## Results

WBC count

**Figure 1:**
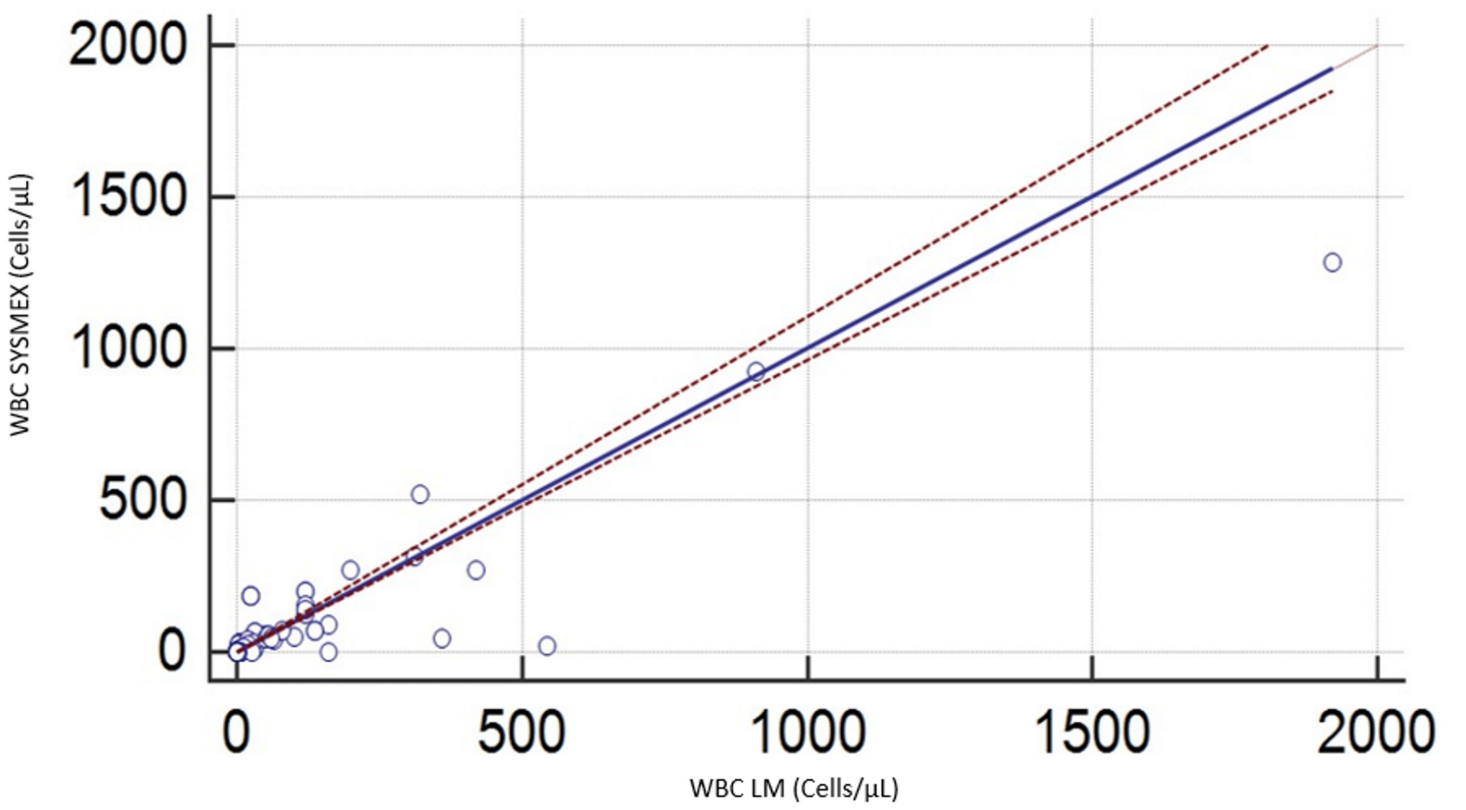
Comparison between the manual technique and Sysmex XN in cerebrospinal fluid for white blood cells WBC: White blood cells; LM: Light microscopy

Our results using the Passing-Bablok regression analysis (Figure 1 and Table 1) suggest an absence of constant and proportional bias between the manual and the automated methods using the Sysmex XN. Table 1 shows a relative standard deviation between -129,86 and 129,86, suggesting a possible absence of concordance between the two methods.

**Table 1:** Passing-Bablok analysis results for the manual and Sysmex XN-1000 methods (red and white blood cells) N: Number of analyzed specimens; WBC: White blood cells; RBC: Red blood cells; CI: Confidence interval; RSD: Relative standard deviation.

	WBC	RBC
N	121	121
Regression equation	Y=0.000000+ 1.003215 X	Y= 0.000000 + 1.128668X
CI intercept	[-0.05; 0.02]	[-1.56; 0.00]
CI slope	[0.96; 1.11]	[0.88; 1.56]
RSD & 95% CI	66.25 [-129,86; 129.86]	1917.25 [-3757,82; 3757,82]
Cusum test for linearity	0.05	<0.01

**Figure 2:**
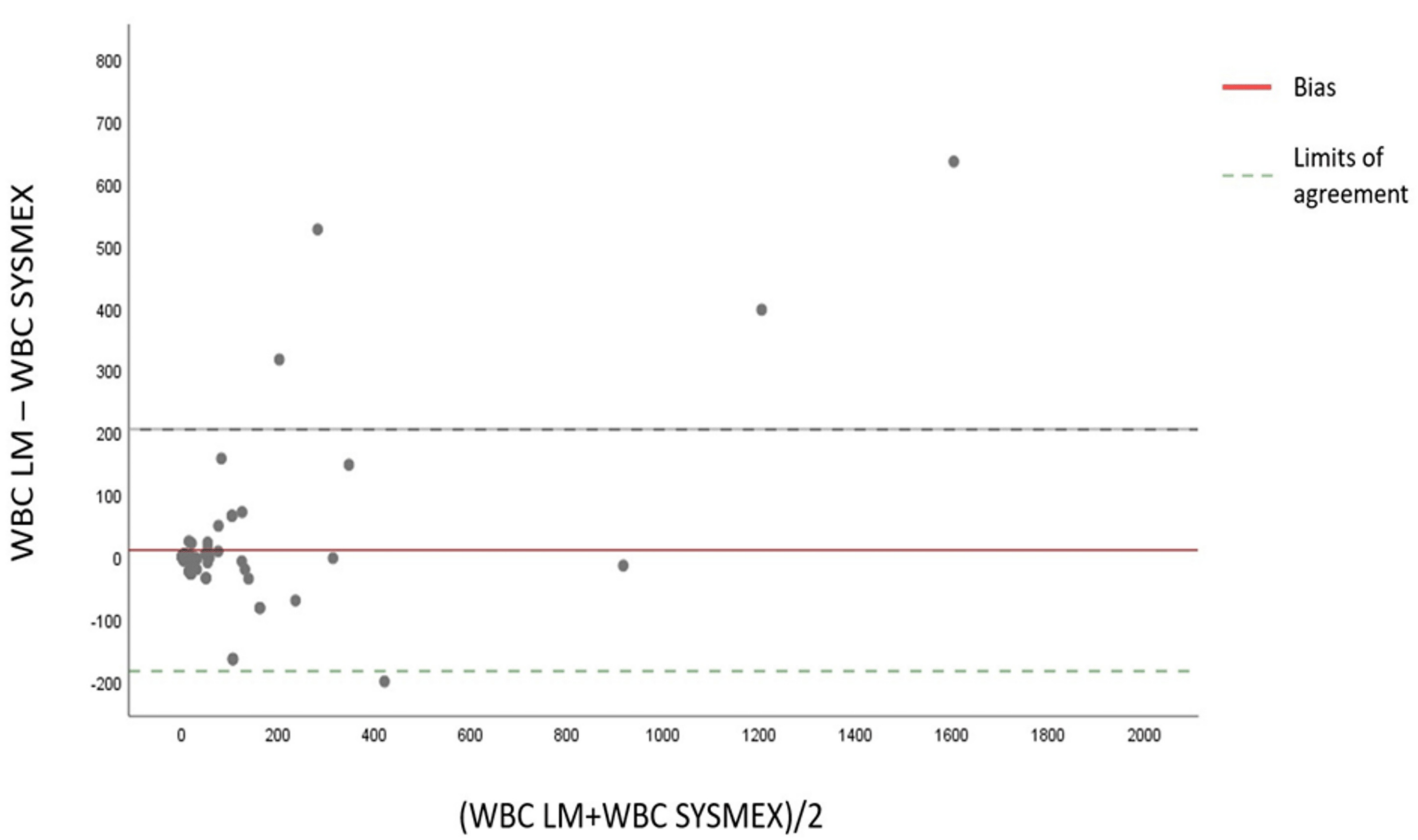
Bland-Altman graph comparing the manual technique and Sysmex XN in cerebrospinal fluid for white blood cells WBC: White blood cells; LM: Light microscopy

In our study, we also used the Bland-Altman plot to evaluate precision in terms of WBC. The graph shows a positive mean bias with a value of 11, suggesting a lack of precision (Figure 2). 

RBC count

**Figure 3:**
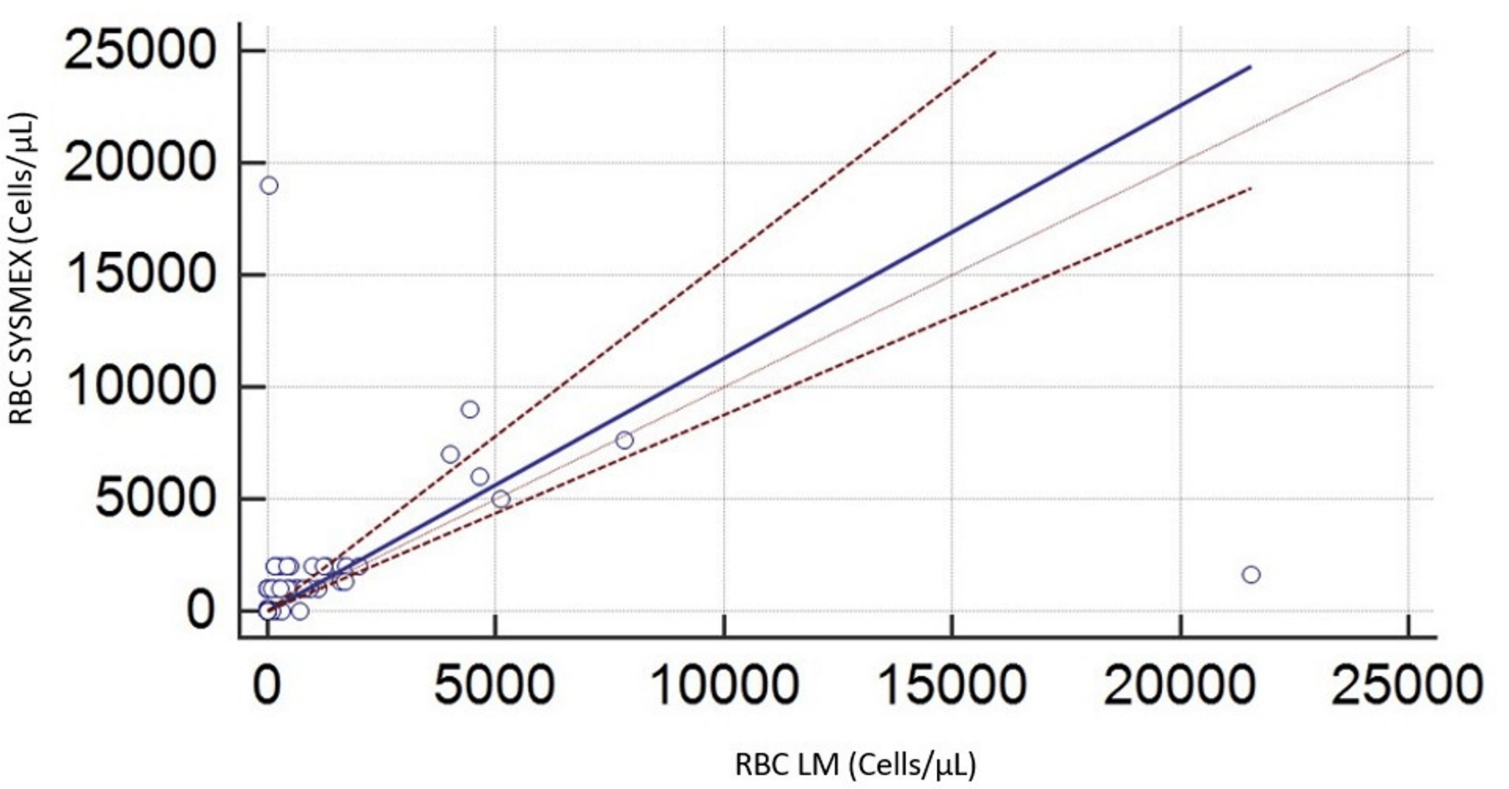
Comparison between the manual technique and Sysmex XN in cerebrospinal fluid for red blood cells RBC: Red blood cells, LM: Light microscopy

**Figure 4:**
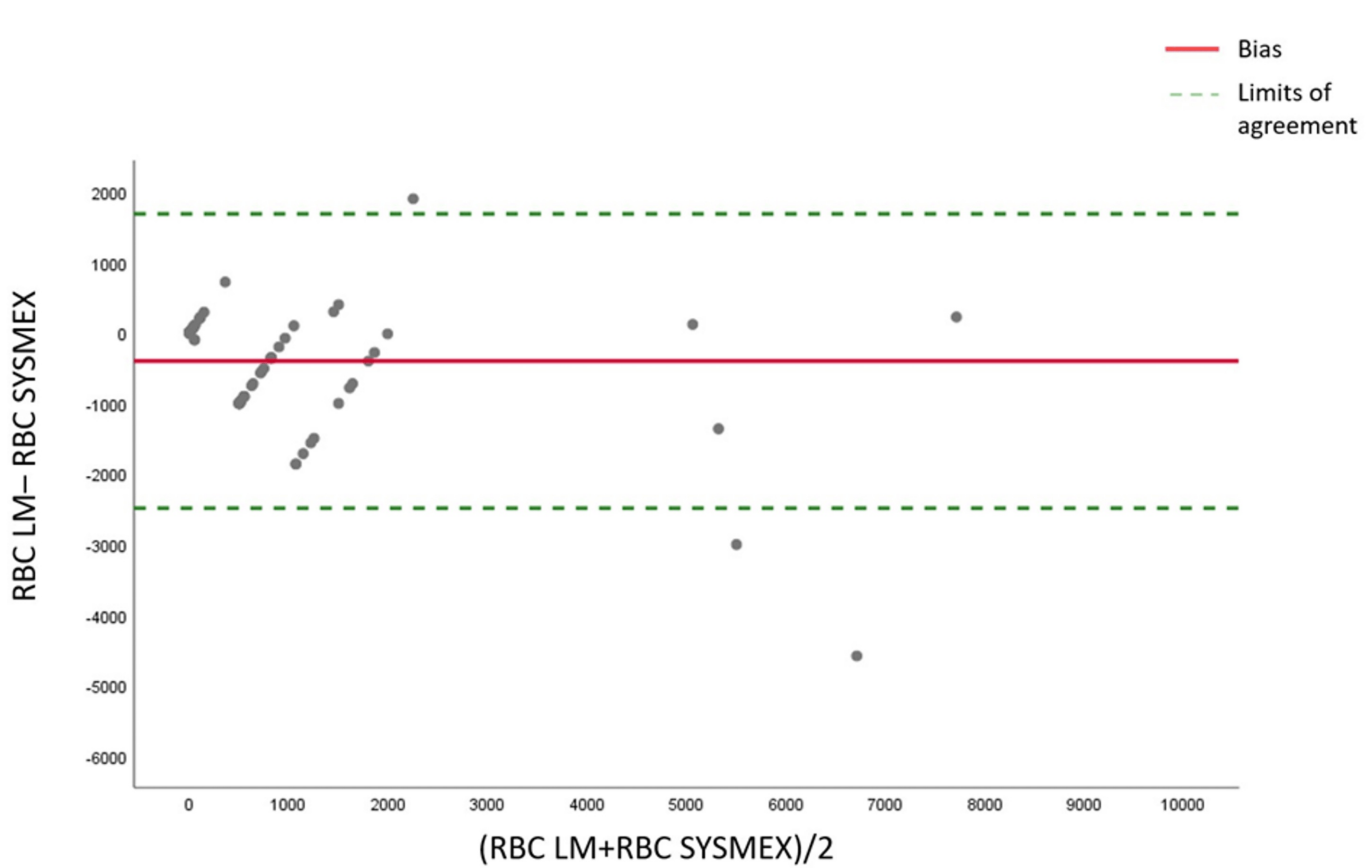
Bland-Altman graph comparing the manual technique and Sysmex XN in cerebrospinal fluid for red blood cells RBC: Red blood cells, LM: Light microscopy

Regarding RBC, the Passing-Bablok regression analysis for RBC counting revealed no evidence of concordance between the two methods, as shown in Figure 3 and Table 1. There was no proportional bias or constant bias, and a large relative standard deviation interval (-3757,82 to 3757,82) was noted. Additionally, the Bland-Altman plot indicates a lack of precision, further illustrated in Figure 4 and Table 1.

It is also noted through the analysis of the graphs (Bland-Altman and Passing-Bablok) for the two cell populations (RBC and WBC) that the difference in counting between the two methods (manual and automated) is much more significant for samples that are very rich in cells.

## Discussion

Automated and standardized analysis methods, such as the analysis enabled by the XN analyzer, are an effective way to get a reduction in response time and inter-laboratory variability [12,13]. However, reduction in those two parameters should be associated with the accuracy of results [12]. The first methods ever used to count cells in a given biological fluid were performed by hemocytometric counting chambers. High inter-observer variability and poor reproducibility are the two major limitations of using different manual methods in cell counting [14].

Automatic hematology analyzers are available in hematological laboratories and are primarily designed for cell counting and cell differentiation in blood specimens. Hence, they are not optimal for other biological fluids like the CSF evaluated in our work. This is due to two major factors: a non-suitable detection limit in biological fluids, other than blood, such as CSF, and the different matrices in blood compared to other biological fluids. According to these two main differences, many discordances between blood cell counts from blood and other biological fluids may be attributed to the interference of mesothelial cells, macrophages, and tumor cells with WBC [12,15,16].

In the literature, there are variable results in terms of the agreement between the manual and automated methods. A study carried out in Spain in 2018 analyzed 103 fluid samples, 31 of which were CSF samples. As in our study, automated counting was performed using a Sysmex XN-1000 engine. For manual counting, Fuchs-Rosenthal counting chambers were used. Evaluation of concordance between the two methods, using Passing-Bablok regression and the Kappa index, showed agreement and clinical significance for WBC and RBC counts in CSF samples [9].

In another similar study in Italy, aiming to compare cell analysis results between the automated and manual techniques in CSF samples, the Sysmex XN-body fluid module was used. The results showed evidence of agreement between the two methods but a limitation with regard to total cell count. The reported cutoff for cells would be 5x106 cells/L. This study recommended that it would be advisable to use the manual method through light microscopic examination when cells exceed the mentioned cutoff [15].

Sysmex XE-5000 is another type of cytological analyzer that was evaluated in a Swedish study in 2014. Automated WBC counting in 198 CSF samples, using the body fluid mode on Sysmex XE-5000, was performed for the sake of being compared to the manual method by microscopy. The results of the study showed good correlation between the manual and automated methods although the precision was limited in samples with a low WBC count [17,18].

Hybrid hemocytometers with modules for biological fluid analysis are being developed to provide benefits from automation, including accuracy, precision, and efficiency. However, each laboratory should verify these advantages for each liquid type [13]. Several studies had positive reports on the accuracy and reproducibility of these analyzers, including the Sysmex XN. However, there are several discrepancies in accuracy assessments comparing automatic methods and manual microscopic methods depending on the cell type (WBC or RBC) or liquid type [14]. In our study, the concordance analysis between the manual technique and the automatic method using Sysmex XN did not find any agreement between RBC and WBC counts in CSF.

Moreover, our study relied on a single automated analyzer (Sysmex XN-1000) and specific manual techniques (Fast Read 102® counting chambers), which may not be representative of other automated systems or manual counting methods available in different laboratories. Finally, although we used Passing-Bablok regression and Bland-Altman analysis to evaluate concordance, these statistical methods have their limitations, particularly in terms of their sensitivity to outliers.

Despite these limitations, our findings contribute to the understanding of the differences between manual and automated cell counting methods in CSF analysis. Future studies should consider addressing these limitations by employing larger sample sizes, multi-center approaches, and a broader range of counting methods to validate the findings further.

## Conclusions

The manual method for white and red blood cells in cerebrospinal fluid samples is considered as a reference method. This method has a number of limitations, such as dependence on the experience of the examiner, the existence of inter-observer variations, and the long response time. Still, not all studies revealed high agreement between the automated techniques and the results obtained by light microscopy. The accuracy of automatic techniques should therefore be improved.
